# HIV prevalence in severely malnourished children admitted to nutrition rehabilitation units in Malawi: Geographical & seasonal variations a cross-sectional study

**DOI:** 10.1186/1471-2431-8-22

**Published:** 2008-05-21

**Authors:** Susan Thurstans, Marko Kerac, Kenneth Maleta, Theresa Banda, Anne Nesbitt

**Affiliations:** 1 Action Against Hunger Malawi, Lilongwe, Malawi; 2 College of Medicine, University of Malawi, Blantyre, Malawi; 3 Ministry of Health, Malawi

## Abstract

**Background:**

Severe malnutrition in childhood associated with HIV infection presents a serious humanitarian and public health challenge in Southern Africa. The aim of this study was to collect country wide data on HIV infection patterns in severely malnourished children to guide the development of integrated care in a resource limited setting.

**Methods:**

A cross sectional survey was conducted in 12 representative rural and urban Nutrition Rehabilitation Units (NRUs), from each of Malawi's 3 regions.

All children and their caretakers admitted to each NRU over a two week period were offered HIV counselling and testing. Testing was carried out using two different rapid antibody tests, with PCR testing for discordant results. Children under 15 months were excluded, to avoid difficulties with interpretation of false positive rapid test results.

The survey was conducted once in the dry/post-harvest season, and repeated in the rainy/hungry season.

**Results:**

570 children were eligible for study inclusion. Acceptability and uptake of HIV testing was high: 523(91.7%) of carers consented for their children to take part; 368(70.6%) themselves accepted testing.

Overall HIV prevalence amongst children tested was 21.6%(95% confidence intervals, 18.2–25.5%). There was wide variation between individual NRUs: 2.0–50.0%.

Geographical prevalence variations were significant between the three regions (p < 0.01) with the highest prevalence being in the south: Northern Region 23.1%(95%CI 14.3–34.0%), Central Region 10.9%(95%CI 7.5–15.3%), and Southern Region 36.9%(95%CI 14.3–34.0%).

HIV prevalence was significantly higher in urban areas, 32.9%(95%CI 26.8–39.4%) than in rural 13.2%(95%CI 9.5–17.6%)(p < 0.01).

NRU HIV prevalence rates were lower in the rainy/hungry season 18.4%(95%CI 14.7–22.7%) than in the dry/post-harvest season 30.9%(95%CI 23.2–39.4%) (p < 0.001%).

**Conclusion:**

There is a high prevalence of HIV infection in severely malnourished Malawian children attending NRUs with children in urban areas most likely to be infected. Testing for HIV is accepted by their carers in both urban and rural areas. NRUs could act as entry points to HIV treatment and support programmes for affected children and families. Recognition of wide geographical variations in childhood HIV prevalence will ensure that limited resources are initially targeted to areas of highest need.

These findings may have implications for the other countries with similar patterns of childhood illness and food insecurity.

## Background

Malnutrition and Human Immunodeficiency Virus (HIV) infection are intimately linked and together present a serious humanitarian and public health challenge in Southern Africa[1]. To date country programmes to address severe malnutrition in childhood have been largely separate from HIV/AIDS treatment and care initiatives [2]. Some malnutrition manuals in current use continue to advocate avoidance of HIV testing except in specific clinical situations[3]. However, with the introduction of ARV (Antiretroviral) therapy there is increasing need for integration of services[4] and targeting on both a population and individual basis.

Malawi is one of many countries affected by the HIV epidemic and malnutrition. According to the 2004 Malawi Demographic and Health (DHS) Survey [5], the overall national adult (15–49 years) prevalence rate of HIV is 11.8%. There are marked in-country geographical variations, as a result of differences in socioeconomic and cultural status:

~ Regional HIV prevalence rates:

- Northern: 8.1%

- Central: 6.5%

- Southern: 17.6%

~ Rural-urban HIV prevalence rates:

-Urban: 17.1%

-Rural: 10.8%

Childhood malnutrition is endemic in Malawi. In the same 2004 DHS survey, 48% of children under 5 were stunted (low height-for-age/chronically malnourished) and 5% were wasted (low weight-for-height ~ acutely malnourished). Severe acute malnutrition, SAM (-3 standard deviations or less from the median weight for height) affects 0.9%–3.2% of children. There are geographical variations in SAM prevalence, with highest (2.6%) prevalence in the Southern region. As 18% of Malawi's approximate 11 million population are less than 5 years old, this represents large numbers of affected and at-risk children [6].

There are a total of 92 Ministry of Health and Christian Health Association of Malawi (CHAM) NRUs across Malawi, 70 of which are currently being supported by non governmental organisations (NGOs) in the implementation of the national protocols for the treatment of severe malnutrition through the continued provision of training and supervision. At the time of this study the standard Malawi National protocols in place were adapted from the WHO '10 step' guidelines which are inpatient based and use F75 and F100 milks. All supported NRUs were working with staff trained to the same protocol, however there were wide variations in NRU outcomes with some of the best resourced urban NRU's having the worst outcomes. It was not clear whether this was due to sub-optimal care in the urban NRU's or whether the children presenting there had more complex underlying clinical problems and specifically whether these were HIV related.

Since HIV infection directly affects all of the principle NRU outcomes (nutritional cures; deaths; rates of weight gain)[7,8], the background rates of HIV prevalence in children being treated for SAM need to be taken into account when assessing the performance of an individual NRU. International guidelines for the treatment of SAM, which originally discouraged HIV testing [3], the limited availability of HIV test reagents and the perception that carers would be unlikely to consent to testing contributed to delays in exploring the linkages. An anonymous linked study in an urban referral NRU in the Southern region of Malawi [8] found a HIV prevalence of 34% among severely malnourished children, however there was no countrywide data other than the known variation in adult prevalence

The aim of this study was to quantify the extent of and geographical distribution of child HIV infection in a representative range of NRUs in Malawi and to assess the acceptability of HIV testing to carers of severely malnourished children in both urban and rural settings

## Methods

Study protocols were reviewed and approved by the Malawi College of Medicine Research and Ethics Committee (COMREC).

A cross sectional study was carried out; data was collected in two separate survey rounds to assess seasonal HIV prevalence variation. The post harvest/dry season survey took place in June 2004, and the rainy/hungry season survey in February 2005. Each survey studied NRU admissions over a 2 week period. Every child admitted was assessed for eligibility to take part.

The sample size of children to be tested was calculated using an α (significance) of 95% and a power of 80% to detect a significant difference between the following:

- Rainy (hungry) season prevalence vs dry season prevalence

- Rural vs urban prevalence

- Northern vs Central vs Southern region prevalence

The lowest prevalence was assumed to be the same as the adult HIV baseline prevalence (11%) and a clinically relevant difference was taken to be twice this value (22%). This gave 200 patients per group at each comparison. To confirm these assumptions were adequate, power calculations were repeated at study completion. These showed, that with an α of 95%, all but one comparison was adequately powered with the numbers that were recruited:

- 83% power for the rainy vs dry season comparison

- 99.9% for the rural vs urban comparison

- 72.9% for the central vs northern region comparison

- 100% for the central vs southern region comparison

- 52.1% for the northern vs southern region comparison

**Figure 1:**
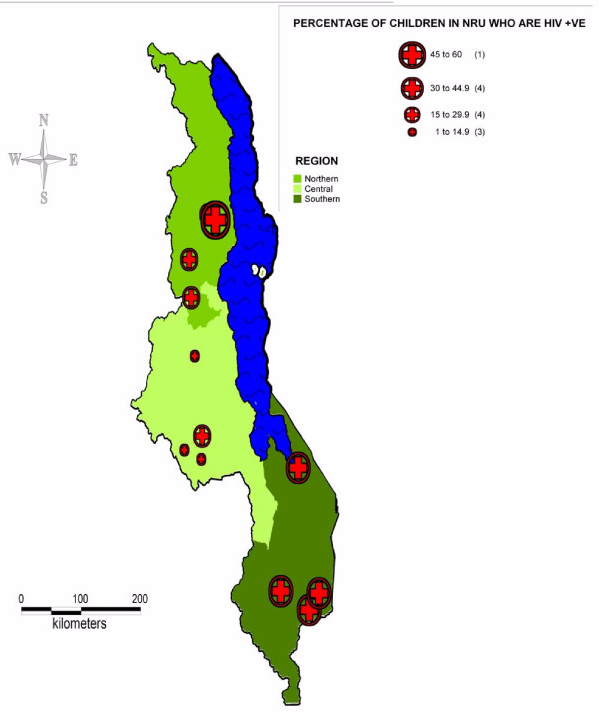
Map of Malawi showing study NRU sites.

The study was conducted in a total of 12 NRUs located throughout each of Malawi's 3 regions (see map, figure 1). Four NRU's were studied in each region: the NRU attached to the main regional referral centre, one other urban NRU and two rural NRU's selected on the basis of the previous years admission data as being representative of their region.

Data was collected by a team of 14 nurses recruited for the purpose of study. The team received specialist training on HIV counselling and testing. This skills/knowledge capacity building was a deliberate study feature, planned to be of long term benefit to study staff and future patients at their regular workplaces.

A study nurse was stationed at each NRU. They informed caretakers of all eligible children about the study. Informed voluntary consent was obtained prior to participation. Children under 15 months were not recruited because of the difficulty in interpretation of results with the antibody-based spot tests that were used. This age cut-off was chosen in line with a 2004 WHO Consultation on the Management of Severe Malnutrition[9]. All caretakers, including those of children < 15 m were offered voluntary counselling and testing (VCT).

HIV testing was carried out in line with recommended national guidelines using Determine and Unigold antibody-based spot tests [10]. Both tests had to agree positive for a positive result to be given. Where the results were discordant, though normal Malawi guidelines recommend a third test as a tie breaker. For a definitive study result, in such cases, a blood sample was sent for HIV viral polymerase chain reaction (PCR) to determine true status.

When a child was found to be HIV positive, the carer was counselled, following national guidelines. Referrals for ongoing care were made, after discussion with caregivers, local clinicians and community services. These included referrals to treatment centres providing ARV and opportunistic infection treatment; community home based care groups; prevention of mother to child transmission initiatives; voluntary counselling and testing centres; orphan care centres; and palliative care services. Antiretroviral therapy for children was not available during the first round of the study and was in limited provision for adults. During the second round it was available at 2 sites.

Data from the study was entered using Microsoft ^® ^Office Excel 2003 and analysed using EpiInfo 3.3.2 ™ (CDC, Atlanta, USA). Odds ratios were calculated and Chi2 tests of significance performed using the StatCalc utility.

Mapping was with Mapinfo 8.5 Professional software (Mapinfo Corp, USA).

## Results

In total 570 children were eligible for the study.

Uptake of HIV testing was high: 523 (91.7%) carers consented to take part in the survey. Results were available for 522 children, 246 (47.6%) girls and 271 (52.4%) boys. 136 children were enrolled during the quiet post-harvest season and 386 during the rainy/'hungry' season round of data collection.

Uptake of testing amongst carers themselves was also high: 368 (70.6%) of carers of enrolled children were themselves tested. The main reasons given for not testing included the need to consult husbands or feeling that they were not sick and therefore did not want to be tested. Where children were positive but the mother had refused testing, the implications of the child's results were clearly explained. PCR testing was only required for 6 children.

Overall, HIV prevalence amongst the children tested was 21.6% (95% confidence intervals, 18.2–25.5%). There were no significant male-female differences (p = 0.11), with a prevalence of 24.4% HIV positive amongst boys, (95% CI 19.4–29.9%) and 18.7% amongst girls (95% CI 14.0–24.1%).

Seasonal and geographical HIV prevalence variations were marked and were statistically significant. These are shown in Table 1.

**Table 1:** Seasonal and geographical HIV prevalence variations.

	Variables	% HIV positive (95% CI)	Odds ratios (95% CI)
Seasonal variations in NRU HIV prevalence Chi2 test: p < 0.01	Rainy/hungry season (n = 386)	18.4% (14.7–22.7%)	1 (reference)
	Dry/post-harvest season (n = 136)	30.9% (23.2–39.4%)	1.98 (1.24–3.17)
Rural-Urban variations in NRU HIV prevalence Chi2 test: p < 0.01	Rural (n = 296)	13.2% (9.5–17.6%)	1 (reference)
	Urban (n = 225)	32.9% (26.8–39.4%)	3.23 (2.04–5.12)
Regional variations in NRU HIV prevalence Chi2 test: p < 0.01	Northern (n = 78)	23.1% (14.3%–34.0%)	2.44 (1.21–4.92)
	Central (n = 265)	10.9% (7.5%–15.3%)	1 (reference)
	Southern (n = 176)	36.9% (29.8–44.4%)	4.75 (2.83–8.01)

- The odds of a severely malnourished child admitted during the dry/post harvest season being HIV positive are almost twice as great as those of a child admitted during the rainy/hungry season.

- Children in urban NRUs have over 3 times the odds of being HIV positive as do children in rural NRUs.

- Regional variations are shown in table 1, with exact locations and corresponding HIV prevalence at individual study sites mapped in Figure 1. The Southern region is both the most densely populated region of Malawi and has the highest HIV prevalence. A child in a Southern region NRUs has almost 5 times the likelihood of being HIV positive than a child in a Central region NRU.

Table 2 shows HIV and clinical type of malnutrition according to presence or absence of oedema (kwashiorkor and marasmus respectively). We had these results for 477/523 patients tested and present data only for these since there is no reason to believe that this is a biased sample and unrepresentative of the whole group.

**Table 2:** HIV and clinical type of malnutrition according to presence or absence of oedema (kwashiorkor and marasmus respectively).

Type of Malnutrition	HIV negative	HIV positive	Totals
Kwashiorkor (oedematous malnutrition)	n = 348 *(87.2% of kwashiorkor) *(89.9% of HIV negative)	N = 51 *(12.8% of kwashiorkor) *(57.3% of HIV positive)	399 *(100% of kwashiorkor)*
Marasmus	n = 39 *(50.6% of marasmus) *(10.1% of HIV negative)	n = 38 *(49.4% of marasmus) *(42.7% of HIV positive)	77 *(100% of marasmus)*
Totals	n = 387 (100% of HIV negative)	n = 89 (100% of HIV positive)	477 *(100%) *(100%)

Overall, children with HIV had more kwashiorkor (57.3%) than marasmus (42.7%). This was mainly because kwashiorkor is the prevalent type of malnutrition in Malawi – 83.6% of total patients presented with kwashiorkor. At individual level however, patients with marasmus were more likely to be HIV positive (49.4% of marasmic patients had HIV) than patients with kwashiorkor. (12.8% of kwashiorkor patients had HIV)

## Discussion

### Test acceptability

This study demonstrates a high rate of uptake of HIV testing of > 90% by carers for children with SAM in both rural and urban NRU's in Malawi following counselling by specially trained nurses. The uptake rate by carers for personal testing > 70% is also higher than anticipated. Both findings counter earlier perceptions that families would be reluctant to participate in testing programmes. A similarly high uptake rate had been previously recorded in a paediatric inpatient prevalence study in an urban Malawian hospital, where overall HIV prevalence was found to be 18.9%, 40% of whom had a diagnosis of malnutrition [11].

At the time the first phase of the study was being conducted ARV treatment programmes for adults were beginning to roll out, they were increasingly well established in regional centres by the time of the second phase. Support services, including home based care, nutrition supplements, PMTCT programmes and cotrimoxazole prophylaxis were becoming more widely available, along with community awareness that case identification was the key to programme access. It is likely that all these factors along with the opportunity to talk privately with trained counsellors who were not normally resident in the local community contributed to the high uptake rates.

### Regional variations

The geographical prevalence patterns of HIV in the NRU's we surveyed reflect the adult regional and urban/rural variations recorded in the 2004 Malawi Demographic and Health Survey. This consistency confirms the validity of our data, which showed a five fold difference between the highest rates in the Southern Region urban NRU's and lowest rates in Central Region rural NRU'S. These findings have important practical implications:

- Firstly there is a need for efficient resource utilization. Knowledge of HIV prevalence rates along with an understanding of concomitant opportunistic infections in HIV disease contributing to SAM, means that agencies can target and allocate food supplies and medication more accurately, NRU's with high HIV prevalence are likely to need larger food allocations as infected children are likely to stay longer in the programme, similarly they are likely to need greater access to antiretrovirals, cotrimoxazole and antifungal reagents than areas of low prevalence.

- Secondly the wide variation in HIV prevalence rates is likely to explain, at least in part, the wide variation in NRU outcomes. Rural NRU's have generally had lower mortality rates and higher cure rates than the urban NRU's[12]. Whilst to date this has been attributed to overcrowding and poor clinical care, the contribution made by coexisting HIV in SAM to high mortality and morbidity rates cannot be overlooked.

- Thirdly, it shows the importance of taking international outcome standards in context. Although Sphere standards[13] for therapeutic feeding programmes previously stated that mortality rates should not be above 10% and cure rates should be above 80%, there has been a recognition that these standards may not be attainable in areas of high HIV prevalence where there is no access to HIV treatment programmes.

### Seasonal variations

Although numerically there were more HIV positive children presenting in the rainy season, proportionately more HIV positive children are admitted in the dry season. We think it likely that these differences reflect changes in primary malnutrition rates. In contrast, HIV is a chronic condition, vertically acquired by most children, and thus prevalence and incidence are relatively constant throughout the year.

- In the dry (post-harvest) season, food is relatively plentiful, NRU admissions fall and only children with underlying disease become sick and malnourished. Hence the high percentage of children with underlying HIV in dry season.

- In the pre-harvest rainy (hungry) season, food shortages, and common childhood illnesses (e.g. malaria and diarrhoeal disease) are major problems, affecting very large numbers of otherwise healthy children. As a percentage of admissions, HIV therefore decreases. However, absolute numbers of children with HIV increase because these children are also affected by the very same factors – and are indeed even more vulnerable than others due to their higher baseline metabolic and nutrition needs [1,9,14].

### Service implications

As HIV treatment services for children develop, routine access to high quality VCT in all NRU's, with adequate supplies of test materials and well trained staff would ensure that children received timely and appropriate clinical interventions. The findings from this study indicate that there should be a move in international guidelines towards promotion of paediatric HIV testing where it can result in access to effective HIV prevention, treatment and care services. Guidelines would need to be adapted locally to address the complex social and holistic needs of affected children and their families. This study reinforces the need for the provision of integrated clinical care programmes linking therapeutic feeding and CTC programmes with HIV treatment programmes.

### Limitations of the study

The authors recognise the limitations of the study:

First, the short sampling period of two episodes of two weeks may have resulted in an over representation of HIV positive children due to the longer period of time they tended to spend in the NRUs.

Second, though our main comparisons were adequately powered in terms of individual patients (see methods), the relatively small number of NRUs that we sampled from risked a selection bias. We do not believe that this happened in practice since our prevalence distributions reflect prevalence's from much larger and more robust adult surveys[5].

Lastly, we did not in this study directly assess the clinical presenting features or mortality implications of having HIV. This has been reported elsewhere[15,16], and we wished to focus on showing the magnitude of the problem at public health level.

## Conclusion

HIV prevalence amongst severely malnourished children in Malawian NRUs is high (26%). The high level of acceptability of HIV testing (> 90%) creates many opportunities to strengthen the services for both conditions.

HIV counselling and testing should be offered routinely to all children and carers presenting to NRUs in Malawi. In settings where the implementation of routine testing is challenging, a starting point could be the areas of highest HIV prevalence. NRUs could provide holistic, family centred care and provide entry points to both HIV prevention, treatment and care programmes, many of which require knowledge of HIV status.

These principles of service integration and geographical targeting are relevant to other countries in the region faced with the dual problems of HIV and food insecurity.

## List of abbreviations

AAH: Action Against Hunger; AIDS: Acquired immune deficiency syndrome; ARV: Antiretroviral; CHAM: Christian Health Association of Malawi; CI: Confidence Interval; COM: College of Medicine; COMREC: College of Medicine Research and Ethics Committee; CTC: Community-based Therapeutic Care; HIV: Human Immune Deficiency Virus; MACRO: Malawi AIDS Resource Organisation; NGO: Non Governmental Organisation; NRU: Nutrition Rehabilitation Unit; PCR: Polymerase Chain reaction; QECH: Queen Elizabeth Central Hospital; SAM: Severe Acute Malnutrition; UNICEF: United Nations Children's Emergency Fund; VCT: Voluntary Counselling and Testing; WHO: World Health Organisation.

## Competing interests

The authors declare that they have no competing interests.

## Authors' contributions

ST participated in the design of the study, organised the implementation and supervision, supervised data collection and entry, and compiled the initial draft paper. MK conceived the initial design of the study, participated in the field supervision and performed final statistical analysis. TB and AN proposed and obtained consent for the study, AN participated in the study supervision and completion of final manuscript. KM performed initial statistical analysis. All authors contributed to, read and approved the final manuscript.

## Pre-publication history

The pre-publication history for this paper can be accessed here:


